# Palliative Care in Intensive Care Units: Nurses' Perspectives on Challenges and Strategies

**DOI:** 10.1111/nicc.70240

**Published:** 2025-12-19

**Authors:** Daniela Filipa Almeida da Cunha, José Carlos Fernandes Alves, Ana Jorge Santos Marques, Rui Filipe Sá Santos, Liliana Andreia Neves da Mota

**Affiliations:** ^1^ Center for Interdisciplinary Research in Health, Faculty of Health Sciences and Nursing Catholic University of Portugal Porto Portugal; ^2^ Porto Nursing School University of Porto Porto Portugal; ^3^ Intensive Care Unit Local Healthcare Unit of Braga Braga Portugal; ^4^ Intensive Care Unit Local Healthcare Unit of Gaia/Espinho Porto Portugal; ^5^ Neurosurgical Ward Local Healthcare Unit of Santo António Porto Portugal; ^6^ Northern School of Health Portuguese Red Cross Oliveira de Azemeis Portugal

**Keywords:** critical care, nurses, palliative care, patient comfort

## Abstract

**Background:**

The integration of palliative care into intensive care units is increasingly recognised as essential to ensuring quality end‐of‐life care, yet persistent barriers continue to challenge its implementation. Nurses, as continuous bedside providers, are central to delivering comfort, but their perspectives remain underexplored in the Portuguese context.

**Aim:**

To explore the challenges and strategies identified by intensive care nurses in the provision of palliative care, guided by Kolcaba's Comfort Theory.

**Study Design:**

A qualitative descriptive design was adopted. Data were collected through a broader questionnaire on palliative care that included two open‐ended questions. Written responses were analysed using Bardin's content analysis, with interpretation informed by Kolcaba's Comfort Theory.

**Results:**

From 52 intensive care nurses, five categories emerged: physical comfort (symptom control and proportionality of interventions), psychospiritual comfort (emotional and spiritual support), sociocultural comfort (family involvement and shared decision‐making), environmental comfort (privacy and humanisation of care) and organisational factors (training needs, institutional resistance and protocols). Nurses highlighted barriers such as therapeutic obstinacy, insufficient training and organisational constraints, while proposing strategies centred on communication, teamwork and education.

**Conclusions:**

Kolcaba's Comfort Theory provided a meaningful lens to interpret the multidimensional nature of comfort in intensive care palliative care. The findings extend understanding of how nurses perceive and address structural and cultural barriers, contributing to theory‐informed nursing knowledge.

**Relevance to Clinical Practice:**

Grounding practice in nurses' perspectives and comfort theory may enhance education, organisational policies and models of care, promoting a more consistent integration of palliative care in intensive care units.

**Impact Statements**

- What is known about the topic
  ○ A significant proportion of ICU patients die in these settings, yet palliative care integration remains limited.
  ○ Comfort, as conceptualised by Kolcaba, encompasses physical, psychospiritual, sociocultural and environmental dimensions that are essential at the end of life.
  ○ Barriers to implementing palliative approaches in ICUs include therapeutic obstinacy, communication difficulties and institutional resistance.
- What this paper adds
  ○ Identifies ICU nurses' perceptions of both barriers and strategies to provide palliative care, highlighting their frontline role in recognising comfort needs.
  ○ Extends Kolcaba's Comfort Theory by demonstrating the importance of organisational integrity as a transversal determinant of comfort in ICUs.
  ○ Offers practical directions for protocol development, multi‐professional training and humanisation initiatives to improve end‐of‐life care in critical care environments.

## 1 Introduction

Intensive care units (ICUs) represent highly technological environments primarily designed to provide life‐sustaining treatments for critically ill patients. However, the increasing recognition that not all critically ill patients will benefit from curative interventions has prompted a paradigm shift towards integrating palliative care principles within these settings. The European Society of Intensive Care Medicine (ESICM) recently developed comprehensive evidence‐based guidelines emphasising that palliative care for critically ill adults should optimise patient‐centred care, improve outcomes for relatives and support ICU staff in delivering compassionate and effective end‐of‐life care [1]. This integration represents a fundamental challenge to traditional ICU culture, which has historically been dominated by a biomedical model focused on cure and technological intervention.

The provision of palliative care in ICUs encompasses a complex array of clinical, emotional, spiritual and organisational dimensions that extend beyond symptom management to address the holistic needs of patients and families facing life‐limiting illnesses [2]. Healthcare professionals, particularly nurses who provide continuous bedside care, are positioned at the intersection of these competing paradigms and face unique challenges in delivering comfort‐focused care within technology‐intensive environments [3]. Recent systematic reviews have highlighted persistent barriers to quality end‐of‐life care in critical care settings, with nurses identifying multiple obstacles that have remained largely unchanged over decades [4].

Contemporary research demonstrates that barriers to palliative care in ICUs are multifaceted, encompassing organisational, cultural and individual factors. An integrative review by Rubbai et al. [5] identified persistent challenges including inadequate training, communication difficulties and institutional resistance to palliative care integration. Similarly, international studies reveal that ICU nurses worldwide face comparable challenges in navigating the complex intersection of curative and palliative approaches [6].

Despite growing international recognition of the importance of palliative care integration in critical care settings, limited research has explored the specific perspectives of ICU nurses regarding the challenges they encounter and the strategies they employ when providing palliative care. This gap in understanding is particularly pronounced in the Portuguese healthcare context, where cultural, organisational and professional factors may influence the delivery of end‐of‐life care in ways that differ from other European healthcare systems.

Katharine Kolcaba's Theory of Comfort provides a comprehensive theoretical framework for understanding the multidimensional nature of comfort in healthcare settings [7]. The theory conceptualises comfort as the immediate desirable outcome of nursing care, experienced across four contexts (physical, psychospiritual, sociocultural and environmental) and three forms (relief, ease and transcendence). This theoretical lens offers particular relevance to palliative care in ICU settings, where the primary goal shifts from cure to comfort and quality of life.

The integration of palliative care principles in ICUs requires nurses to navigate complex ethical, clinical and interpersonal challenges while maintaining their professional commitment to patient advocacy and holistic care. Understanding these challenges and the strategies nurses develop to address them is essential for developing evidence‐based approaches to improve palliative care delivery in critical care environments, particularly in light of recent international guidelines emphasising the need for structured training and improved family‐centred care approaches [1].

Evidence from systematic reviews indicates that while barriers to palliative care in ICUs are well documented internationally, effective interventions remain limited and context dependent [8]. The ESICM guidelines particularly emphasise early integration of palliative care, standardised symptom assessment tools, communication training for ICU staff and the importance of family‐centred care interventions, highlighting areas where nursing perspectives are crucial for successful implementation [1].

To our knowledge, this study represents the first comprehensive qualitative exploration of ICU nurses' perspectives on palliative care challenges and strategies within the Portuguese healthcare context. By employing Kolcaba's Theory of Comfort as an organising framework, this research aims to provide novel insights into the multidimensional nature of comfort provision in ICU settings and contribute to the limited body of knowledge regarding palliative care integration in Portuguese critical care environments.

## 2 Background/Justification for Study

International evidence shows that early integration of palliative care in intensive care units improves symptom control, reduces admissions and length of stay and enhances family satisfaction with end‐of‐life care [9]. Yet, its implementation remains hindered by insufficient training, limited institutional support and cultural resistance to shifting from a curative to a comfort‐oriented model [10]. These barriers are especially relevant for nurses, who must balance life‐sustaining interventions with the provision of comfort in technology‐driven environments [11]. In Portugal, research on this integration is scarce, particularly regarding nurses' perspectives, making it essential to explore their experiences to inform practice, guide policy and strengthen education while contributing culturally specific insights to the international evidence base.

In Portugal, intensive care is mainly delivered within the publicly funded National Health Service. Despite significant expansion in recent years, ICU bed capacity per capita remains below the OECD average, and marked geographic heterogeneity persists across regions. Nurse staffing is frequently constrained, and the absence of rehabilitation nursing in many ICUs has been reported [12]. These resource limitations combine with high occupancy rates, which frequently exceed 85%, further challenging the integration of palliative approaches. Moreover, cultural norms give families a central role in end‐of‐life decision‐making, which can facilitate shared care planning but also complicate decision processes. These contextual characteristics shape how palliative care is negotiated in Portuguese ICUs and are essential for understanding the transferability of our findings.

Kolcaba's Comfort Theory conceptualizes comfort as an immediate, desirable outcome of nursing care that occurs across four contexts—physical, psychospiritual, sociocultural, and environmental—and in three forms: relief, ease, and transcendence [7]. This multidimensional framework is particularly well suited to intensive care, where patients and families simultaneously face physical symptoms, emotional and spiritual burdens, communication challenges, and environmental stressors. Previous studies have demonstrated its usefulness in guiding holistic care interventions and evaluating comfort outcomes in diverse clinical settings, including oncology, critical care, and palliative care [11, 13, 14, 15]. The theory was selected because it provides a holistic lens through which the complexity of palliative care in ICUs can be interpreted, moving beyond a focus on symptom control to encompass relational, cultural, and organizational aspects of care. Recent scholarship also highlights that theoretical frameworks such as Comfort Theory strengthen the conceptual coherence of palliative care research and facilitate the development of practice models applicable to technology‐driven environments like ICUs [11, 14]. By grounding our analysis in this framework, we sought to ensure conceptual rigor and extend its application to the critical care context.

## 3 Aims and Objectives/Research Questions/Hypotheses

The aim of this study was to explore the challenges and strategies identified by intensive care nurses in the provision of palliative care within intensive care unit settings. Accordingly, the research question guiding this study was: “What challenges and strategies do intensive care nurses recognize in the provision of palliative care?”.

## 4 Design and Methods

A qualitative descriptive design was employed within an interpretive paradigm to explore intensive care nurses' perceptions of palliative care provision. The study was reported in accordance with the Consolidated Criteria for Reporting Qualitative Research (COREQ) checklist [16].

The qualitative analysis was conducted by two researchers (Author 1, MSc, PhD candidate, nurse researcher and palliative care nurse specialist; and Author 2, MSc, PhD candidate, nurse researcher and critical care nurse specialist) with supervision. Both had prior training and experience in qualitative content analysis. Their professional backgrounds and interest in palliative care were acknowledged as potential influences on the interpretive process, and reflexive discussions were used to minimise bias. The analytical process was additionally supervised by Author 5 (PhD, senior researcher with extensive experience in qualitative methodologies and palliative care), ensuring methodological rigour and enhancing the credibility of the findings.

Participants were aware that the study was conducted by nurse researchers specialised in critical care and palliative care, with no prior personal relationships with them.

## 5 Setting and Sample

The study was conducted in adult intensive care units (ICUs) within the Portuguese National Health Service. These units are highly technological environments designed to provide continuous monitoring and organ support for critically ill patients, but where situations of therapeutic limitation and end‐of‐life decision‐making are also frequent. Nurses working in such settings are constantly exposed to the tension between curative and comfort‐oriented care, making them a key professional group for examining perceptions of palliative care integration.

A non‐probabilistic, convenience sampling strategy was used. Eligibility criteria included the following: (i) being a registered nurse with at least 6 months of professional experience in an ICU; (ii) direct involvement in patient care; and (iii) voluntary agreement to participate in the study.

## 6 Data Collection Tools and Methods

Data were collected through an anonymous online questionnaire distributed electronically between June and August 2025. The questionnaire was developed as part of a broader mixed‐methods study on perceptions of palliative care and included both closed‐ and open‐ended items. For the present article, only the responses to two open‐ended questions were analysed. These items specifically invited participants to describe, in their own words, the challenges and strategies they perceived in the provision of palliative care in the ICU.

Thematic saturation was achieved through iterative analysis of the 52 responses, which generated 2432 words of narrative text. Data saturation was evidenced by the repetition of core themes across participants and the absence of new conceptual insights in the final responses analysed. This corpus size aligns with empirical evidence suggesting that saturation in focused qualitative studies with homogeneous populations can be achieved with similar sample sizes [17, 18]. The breadth of perspectives captured across multiple ICU settings further supported the adequacy of the dataset for comprehensive thematic analysis.

The decision to focus exclusively on these open‐ended responses was therefore methodologically justified on two grounds: (i) alignment with the specific aim of this article, which sought an in‐depth exploration of nurses' perspectives on palliative care; and (ii) the volume and quality of textual data obtained, which ensured sufficient depth for rigorous content analysis guided by Kolcaba's Comfort Theory.

The questionnaire included two open‐ended items designed to elicit nurses' perceptions regarding challenges and strategies in palliative care provision. The exact wording of the questions was originally in Portuguese: (1) ‘In your experience, what are the main challenges you face in providing palliative care in the ICU?’ and (2) ‘What strategies do you consider most effective to promote comfort for patients at the end of life, and for their families and/or caregivers, in the ICU?’ Prior to data collection, the items were pilot tested with three ICU nurses to ensure clarity and relevance.

As the responses were collected through an online survey, no repeat responses were requested, and no field notes were taken. Participants did not validate transcripts or findings (member checking), which may limit confirmability. Nevertheless, credibility was supported by independent double coding, supervisory oversight and reflexive discussions.

## 7 Data Analysis

Responses were analysed using Bardin's [19] content analysis framework. The process followed three stages: (i) pre‐analysis through repeated reading to identify semantic units; (ii) coding and categorisation of themes; (iii) interpretation and synthesis. Kolcaba's Comfort Theory was applied as an interpretive framework to organise and understand the emerging categories across physical, psychospiritual, sociocultural and environmental dimensions, along with organisational factors. Coding was conducted independently by two researchers, and discrepancies were resolved by consensus to enhance credibility. Representative quotes were selected to illustrate key themes.

Coding was performed manually without the use of qualitative analysis software. In addition to the predominant categories, the analysis also considered minority and divergent perspectives, which were preserved and reported to ensure that less common but relevant insights were not excluded. This approach aimed to enhance the credibility and comprehensiveness of the findings.

## 8 Ethical and Institutional Approvals

The study protocol was submitted to the Ethics Committee of the Northern School of Health of the Portuguese Red Cross and received approval under Report No. 011/2025, Code 2025.014, on 1 July 2025, with unanimous consent. Participation was voluntary, and informed consent was obtained electronically prior to completing the questionnaire. Confidentiality and anonymity were ensured at all stages of the research process.

## 9 Results/Findings

A total of 52 intensive care nurses participated in the study. The majority were female (*n* = 41, 78.8%), with ages ranging from 25 to 66 years (*M* = 38.8, SD = 9.6). Professional nursing experience averaged 15.9 years (range: 1–44 years), while ICU‐specific experience averaged 10.6 years (range: 0–40 years). Regarding educational qualifications, 37 participants (71.2%) held a bachelor’s degree and 15 (28.8%) had completed a master’s degree. Twenty‐two participants (42.3%) were specialist nurses and 24 (46.2%) reported prior exposure to palliative care training. The demographic and professional characteristics are summarised in Table S1.

Regarding exposure to end‐of‐life situations in the ICU, most participants reported regular contact with such cases. Eighteen participants (34.6%) encountered end‐of‐life situations 1–2 times, per week, while an equal number (34.6%) reported contact 1–3 times per month. Seven participants (13.5%) reported daily exposure to end‐of‐life situations and two (3.8%) indicated contact 3–5 times per week. Seven participants (13.5%) reported less frequent exposure (less than once per month). Overall, 86.5% of participants reported at least monthly contact with end‐of‐life situations, highlighting their substantial experience with palliative care scenarios in the ICU setting. Sociodemographic and professional data were collected through structured items in the broader questionnaire. These variables were used exclusively for sample characterisation and not for inferential analysis, as the focus of this article is the qualitative content derived from the two open‐ended questions.

This sample size is considered adequate for qualitative content analysis, as it ensured a diverse range of experiences while allowing for thematic saturation to be achieved across the responses. The inclusion of participants from multiple ICUs also enhanced the transferability of findings to comparable critical care contexts.

The content analysis of nurses' responses yielded a comprehensive framework of categories and subcategories that reflect their perceptions of the challenges and strategies in providing palliative care within the ICU. In line with Kolcaba's Comfort Theory, the findings were organised across four key dimensions of comfort—physical, psychospiritual, sociocultural and environmental. In addition to these dimensions, the analysis also identified a transversal organisational category, representing ‘institutional integrity’ and systemic factors that either facilitate or hinder the provision of comfort.

Together, these five categories capture the multidimensional nature of comfort in the ICU setting, encompassing both individual‐level nursing practices and broader institutional contexts. Each category includes subcategories that highlight specific aspects of nurses' experiences and perspectives, illustrated with representative quotations. The following sections present these categories in detail.

### 9.1 Physical Comfort: Symptom Control and Reduction of Invasiveness

Nurses consistently emphasised the centrality of rigorous symptom management and the need to limit futile interventions. Therapeutic obstinacy, unnecessary invasive devices and the lack of protocols for treatment limitation were described as major obstacles. Strategies included effective symptom control, early withdrawal of disproportionate measures and advance care planning.

#### 9.1.1 Symptom Control

9.1.1.1

“Rigorous control of symptoms, especially pain, dyspnea, anxiety, and agitation, with appropriate use of analgesia and sedation” (P6) and a “focus [of] care on promoting comfort, mainly through pain control” (P22) were consistently highlighted, underscoring symptom control as central to comfort and perceived quality of life.

#### 9.1.2 Therapeutic Proportionality

Participants reported that “we often invest in invasive and disproportionate measures, even when prognosis is very poor” (P6), while also calling for “withdrawal of therapeutic obstinacy; suspension of dysthanasia” (P33), supporting the need to avoid futile measures and to promote proportional therapeutic limitation.

#### 9.1.3 Care Planning

9.1.3.1

“Advance care planning, discussing intervention limits in line with the person’s values and wishes” (P6) and an “advance care plan focusing on the person’s will and preferences” (P42) were described as essential to align care with what matters most to the person.

### 9.2 Psychospiritual Comfort: Emotional and Spiritual Support

This dimension emerged strongly, with nurses describing the emotional burden of families and staff, unrealistic expectations of recovery, and the difficulty of transitioning to a palliative approach. Proposed strategies included psychological support, spiritual assistance and preparing families for loss:

#### 9.2.1 Psychological Support

9.2.1.1

“Emotional support interventions (...) psychological support for caregivers and staff” (P11) and “having a psychologist available in the interdisciplinary team” (P29) were viewed as key strategies to reduce anxiety and alleviate suffering.

#### 9.2.2 Spiritual Support

9.2.2.1

The “presence of a spiritual leader if desired” (P19) and the “promotion of silence and chaplain presence” (P48) were emphasised as ways of providing spiritual care that respects beliefs and is essential at the end of life.

#### 9.2.3 Preparation for End of Life

9.2.3.1

Nurses described the “difficulty in preparing the family for a healthy grieving process” (P3) and stressed the importance of “emotional, psychological, and spiritual support; shared decision‐making with family” (P40) to reduce suffering and facilitate healthy grieving.

### 9.3 Sociocultural Comfort: Shared Decision‐Making and Family Integration

Challenges were related to communication difficulties between teams and families, resistance to joint decision‐making and late referral to palliative care. Strategies emphasised structured family conferences, respect for advance directives and family integration.

#### 9.3.1 Therapeutic Communication

9.3.1.1

“Clear, empathetic, and continuous communication with the family” (P6) was considered essential, while “difficulty aligning communication between team, patients, and families” (P11) was identified as a barrier, highlighting the role of empathic communication in aligning expectations and supporting coherent decisions.

#### 9.3.2 Shared Decision‐Making

Ensuring that “families are involved in decisions, which helps reduce distress” (P6), together with the “promotion of advance directives of will” (P14), was described as fundamental to build trust and respect for expressed wishes.

#### 9.3.3 Family Integration

9.3.3.1

A “humanized ICU environment—flexible visits, privacy with family” (P11) and “facilitating visits, including pets and meaningful personal belongings” (P24) were seen as strategies that humanise care and enhance comfort through stronger family integration.

### 9.4 Environmental Comfort: Humanisation and Privacy

Environmental limitations such as noise, lack of privacy and visiting restrictions were frequently noted. Strategies included flexible visiting hours, humanisation interventions (music therapy, aromatherapy) and creating welcoming spaces.

#### 9.4.1 Privacy and Visits

9.4.1.1

“Moments of intimacy and privacy with the family” (P11) and “flexible visiting hours and number of visitors” (P25) were viewed as crucial to uphold dignity and support well‐being.

#### 9.4.2 Environmental Humanization

9.4.2.1

“Silence/music according to the person’s preference” (P27) and the “implementation of music therapy and aromatherapy” (P43) were reported as ways to create calmer environments and contribute to more humanized care.

#### 9.4.3 Adapted Spaces

9.4.3.1

Having “adequate physical space for the family to stay with their relative” (P20) and being “hospitalized in an environment with adequate conditions, silence, and calm” (P39) were described as enabling comfort and sustained family presence.

### 9.5 Organisational Variables: Institutional Integrity

Systemic and cultural barriers were widely reported, including a curative‐dominant paradigm, medical resistance, lack of protocols, insufficient training and late referrals. Strategies included multiprofessional training, clear guidelines, articulation with support teams and institutional accreditation in humanisation.

#### 9.5.1 Professional Training

9.5.1.1

“Lack of specific training in palliative care” (P11) and the view that “communication and training, especially of physicians, should be further invested in” (P25) reinforced the importance of targeted education for safe, humanized care.

#### 9.5.2 Protocols and Guidelines

9.5.2.1

The “absence of protocols” (P10) and the “need for protocol creation” (P31) were repeatedly mentioned, suggesting that clear guidelines are needed to support decision‐making and standardize practice.

#### 9.5.3 Curative Paradigm

9.5.3.1

“Reconciling aggressive ICU approach with palliative philosophy” (P11) and the perception that “ICU culture is strongly focused on treatment and cure” (P22) illustrate how a curative‐dominant culture can hinder palliative care integration.

#### 9.5.4 Referral and Articulation

9.5.4.1

“Late referral to palliative care” (P7) contrasted with the “identification of complex needs and request for specialized professionals” (P42), underscoring the importance of early referral and effective articulation with support teams to ensure continuity and quality.

#### 9.5.5 Accreditation

9.5.5.1

“Accreditation in Humanization brings together many of the necessary measures to improve care” (P51), indicating that institutional accreditation can consolidate essential measures to improve care.

### 9.6 Synthesis

Across categories, nurses highlighted the multidimensional nature of comfort in ICUs, spanning physical, emotional, cultural, environmental and organisational contexts. Barriers such as therapeutic obstinacy, inadequate communication and insufficient training were frequently mentioned, but strategies centred on teamwork, family involvement, humanisation and institutional support. Divergent perspectives such as reports of collaborative medical practices or suggestions that palliative care should be relocated outside ICUs added nuance, underscoring the complexity of integrating palliative care into critical care environments. A coding tree summarising categories, subcategories and representative quotations is provided in the impact statements box above.

### 9.7 Divergent Perspectives and Minority Voices

While most participants identified significant barriers, some nurses reported more positive experiences, particularly regarding medical collaboration:

Some physicians do collaborate well, adjusting invasive measures and prioritizing comfort when appropriate. (P48)

In our unit, we've seen improvements in communication between medical and nursing teams regarding end‐of‐life decisions. (P35)

Additionally, perspectives varied regarding the appropriate setting for palliative care delivery:

Palliative care should begin in the ICU, but patients should then be transferred to calmer units. (P39)

I believe ICUs can provide excellent palliative care if we adapt our practices properly. (P27)

These divergent views highlight the influence of local organisational culture and individual professional attitudes on palliative care integration, suggesting that positive change is possible within existing ICU structures.

## 10 Discussion

This study explored ICU nurses' perceptions of palliative care provision, analysed through Kolcaba's Comfort Theory. The findings identified five categories—physical, psychospiritual, sociocultural, environmental and organisational comfort—that reflect both the challenges of end‐of‐life care in ICUs and the strategies proposed to overcome them. Together, these results illustrate the multidimensional and systemic nature of comfort and highlight the need for theory‐informed approaches to integrate palliative care in critical care environments.

Physical comfort was strongly emphasised, with therapeutic obstinacy, unnecessary invasive procedures and the absence of protocols for treatment limitation identified as key barriers. These findings are consistent with evidence that disproportionate interventions remain common in ICUs and exacerbate patient and family suffering [9, 20]. Strategies such as rigorous symptom control, proportionality of interventions and anticipatory care planning align with the literature demonstrating that symptom management is central to comfort and quality of life [11, 21]. Within Kolcaba's framework, the focus on symptom relief underscores the primacy of physical comfort as a foundation for tranquillity and transcendence [7].

The dimension of psychospiritual comfort highlighted the emotional burden of families and staff, the challenge of managing hope, and the difficulty of accepting the transition from a curative to a comfort‐oriented paradigm. Nurses proposed psychological support, spiritual assistance and preparation for loss as key strategies. These findings echo studies showing that spirituality and emotional support are essential for dignity, resilience and healthier grief processes [13]. Psychospiritual care thus emerges as indispensable in preventing compassion fatigue among staff and fostering transcendence for patients and families [7, 22].

Sociocultural comfort was closely tied to communication and shared decision‐making. Barriers included unclear communication and resistance, particularly among physicians, to adopting a palliative approach. Strategies focused on family conferences, advance directives and family integration into care. These findings are in line with studies that demonstrate the importance of structured communication and family participation in promoting trust, satisfaction and emotional adaptation [11]. Importantly, some nurses described more positive experiences of collaboration, highlighting the influence of organisational culture and professional attitudes on care delivery.

Environmental comfort emerged as another critical dimension. Nurses emphasised that noise, lack of privacy and restrictive visiting policies undermine comfort in the ICU. Proposed strategies included flexible visiting hours, environmental adaptations and humanisation interventions such as music and aromatherapy. Evidence supports the view that the humanisation of the ICU environment contributes to dignity and well‐being [23, 24]. However, some participants argued that ICUs are not suitable for end‐of‐life care, suggesting instead referral to calmer settings. These divergent views reflect ongoing debates about the appropriateness of ICUs for palliative care delivery.

Beyond Kolcaba's four classical contexts, a transversal category of organisational variables emerged, reflecting systemic influences on palliative integration. Nurses reported barriers such as insufficient training, lack of protocols, cultural resistance within medical teams and late referral to specialised support. Strategies included multiprofessional training, institutional protocols, inter‐team collaboration and accreditation in humanisation. These findings align with the concept of institutional integrity [14] and are supported by literature showing that organisational alignment is essential for consistent, high‐quality care [14]. Timely referral and institutional policies have also been shown to improve quality of life and reduce costs by avoiding unnecessary interventions [22, 25, 26].

The use of Kolcaba's Comfort Theory provided a robust framework to structure nurses' perceptions, confirming its relevance in ICU contexts. At the same time, the emergence of the organisational category extends the theory by demonstrating that comfort depends not only on direct care but also on institutional structures and cultures that enable or hinder practice. This highlights the importance of theory‐driven approaches in nursing research and care delivery that address both individual and organisational dimensions.

## 11 Limitations

While this study provides important insights into ICU nurses' perceptions of palliative care, several limitations must be acknowledged. The use of a convenience sample of 52 nurses, though informative, restricts representativeness and transferability to other organisational or cultural contexts. Data collection relied on two open‐ended survey questions, which encouraged broad participation but limited narrative depth compared with interviews or focus groups. Member checking was not conducted, reducing confirmability by excluding participants' validation of interpretations. Coding was performed manually without qualitative software, which may have limited transparency despite independent double coding and consensus procedures. Researchers' backgrounds in intensive and palliative care could also have influenced interpretation, although reflexive discussions and supervisory oversight sought to mitigate bias.

Credibility was enhanced through double coding, supervisory review, reflexivity and a robust theoretical framework, strengthening trustworthiness while identifying areas for methodological refinement in future research.

## 12 Recommendations or Implications for Practice and/or Further Research

The findings of this study carry important implications for intensive care practice. First, they highlight the urgent need for institutional protocols guiding therapeutic proportionality and timely palliative care referral, ensuring consistent, transparent and value‐centred decisions. Second, nurses' emphasis on communication and shared decision‐making underscores the importance of multiprofessional education, equipping ICU teams with skills in symptom management, family engagement and ethical deliberation. Third, strategies to enhance environmental humanisation—including flexible visiting, privacy measures and comfort interventions—should be systematically integrated into ICU models to improve patient and family experiences.

From a research perspective, the study emphasises the need to further explore how organisational culture and institutional integrity shape palliative care integration. Future studies should assess the effectiveness of interventions such as training, standardised pathways and accreditation initiatives, using qualitative or mixed‐methods approaches to capture impacts on patient outcomes, family satisfaction and staff well‐being. Comparative studies across cultural and institutional contexts are also warranted to evaluate transferability and inform international best practices.

## 13 Conclusion

These findings reaffirm the multidimensional nature of comfort and expose the systemic barriers that continue to hinder the integration of palliative care in ICUs. For clinical practice, they underscore the imperative of developing institutional protocols, investing in multiprofessional training, strengthening family‐centred communication and advancing environmental humanisation. For research, they highlight the need to investigate how organisational culture shapes the delivery of palliative care and to evaluate interventions that strengthen institutional integrity and sustainability. By situating ICU nurses' perspectives within Kolcaba's Comfort Theory, this study not only validates the theory's relevance to critical care but also extends it to encompass organisational determinants. Ultimately, it calls for a paradigm shift in intensive care—one that integrates science, humanisation and institutional alignment to ensure that dignity and comfort are safeguarded as fundamental outcomes at the end of life:

## Ethics Statement

Approved by the Ethics Committee of the Northern School of Health of the Portuguese Red Cross, Report No. 011/2025, Code 2025.014, on 1 July 2025.

## Consent

Patient consent was not required for this study, as it did not involve patients. Written informed consent was obtained from all participating nurses.

## Conflicts of Interest

The authors declare no conflicts of interest.

## Supporting information


**Table S1:** Participant characteristics (*n* = 52).

## Data Availability

The data that support the findings of this study are available on request from the corresponding author. The data are not publicly available due to privacy or ethical restrictions.
