# Supplementary material for: Palliative Care in Intensive Care Units: Nurses' Perspectives on Challenges and Strategies
Source: Nurs Crit Care. 2025 Dec 19;31(1):e70240. doi: 10.1111/nicc.70240 (PMC12715749; doi:10.1111/nicc.70240)
Supplement: Supplementary file 1 — Table S1: Participant characteristics (n = 52). [file NICC-31-0-s001.docx]

## ***Table S1. Participant Characteristics (n=52)***

| **Characteristic** | **n (%)** | **M (SD)** | **Range** |
| --- | --- | --- | --- |
| Age (years) | - | 38.8 (9.6) | 25-66 |
| Gender  Male  Female | 11 (21.2)  41 (78.8) | -  - | -  - |
| Years in nursing | - | 15.9 (9.8) | 1-44 |
| Years in ICU | - | 10.6 (9.4) | 0-40 |
| Educational Level  Bachelor’s Degree  Master’s Degree | 37 (71.2)  15 (28.8) | -  - | -  - |
| Specialist Nurse Certification | 22 (42.3) | - | - |
| Prior Palliative Care Training | 24 (46.2) | - | - |
| Frequency of End-of-Life Situations  Daily  3-5 times per week  1-2 times per week  1-3 times per month  Less than once per month  Never | 7 (13.5)  2 (3.8)  18 (34.6)  18 (34.6)  7 (13.5)  0 (0.0) | -  -  -  -  -  - | -  -  -  -  -  - |

Note: M = Mean; SD = Standard Deviation; ICU = Intensive Care Unit
